# High-Throughput Illumina MiSeq Amplicon Sequencing of Yeast Communities Associated With Indigenous Dairy Products From Republics of Benin and Niger

**DOI:** 10.3389/fmicb.2019.00594

**Published:** 2019-04-03

**Authors:** Philippe Sessou, Santosh Keisam, Ngangyola Tuikhar, Mariama Gagara, Souaïbou Farougou, Kumaraswamy Jeyaram

**Affiliations:** ^1^Research Unit on Communicable Diseases, Laboratory of Research in Applied Biology, Polytechnic School of Abomey-Calavi, University of Abomey-Calavi, Abomey-Calavi, Benin; ^2^Microbial Resources Division, Institute of Bioresources and Sustainable Development (IBSD), Takyelpat Institutional Area, Imphal, India; ^3^Central Livestock Laboratory, Niamey, Niger

**Keywords:** *Wagashi*, *Calotropis*, MiSeq amplicon sequencing, internal transcribed spacer, *Kluyveromyces*, *Saccharomyces*, *Sagenomella*

## Abstract

Traditional *Wagashi* cheese and fermented cow milk are among the most popular dairy products appreciated by people from Benin, Niger, and the neighboring region. These products are the main source of protein in the diet of the low-income population in the region. The fermented milk is prepared by spontaneous fermentation without back-slopping. Whereas, the leaf extract of *Calotropis procera* is used for curdling the milk to prepare the soft *Wagashi* cheese. The present study aims to provide in-depth analysis of yeast communities associated with these traditional milk products by high-throughput Illumina MiSeq amplicon sequencing of internal transcribed spacer (ITS) region of fungal rRNA genes. A total of 60 samples, 20 samples of fermented milk each from Benin and Niger, and 20 samples of *Wagashi* cheese from Benin were used for analysis. The metagenomic investigation revealed that *Kluyveromyces marxianus*, *Saccharomyces cerevisiae*, *Candida parapsilosis*, and *Sagenomella keratitidis* were the predominant yeast species present in the traditional milk products. Furthermore, we noticed a high presence of *K. marxianus* (61.1% relative abundance) in the *Wagashi* cheese and *S. cerevisiae* (28.4% relative abundance) in the fermented milk of Niger. The presence of potential pathogenic yeast *C. parapsilosis* and *S. keratitidis* in these African milk products calls for further investigation to assess their safety. The predominant yeast *K. marxianus* and *S. cerevisiae*, recognized with generally regarded as safe (GRAS) status, could be further selected as starter culture along with lactic acid bacteria for developing controlled fermentation processes with enhanced product quality and safety.

## Introduction

**FIGURE 1:**
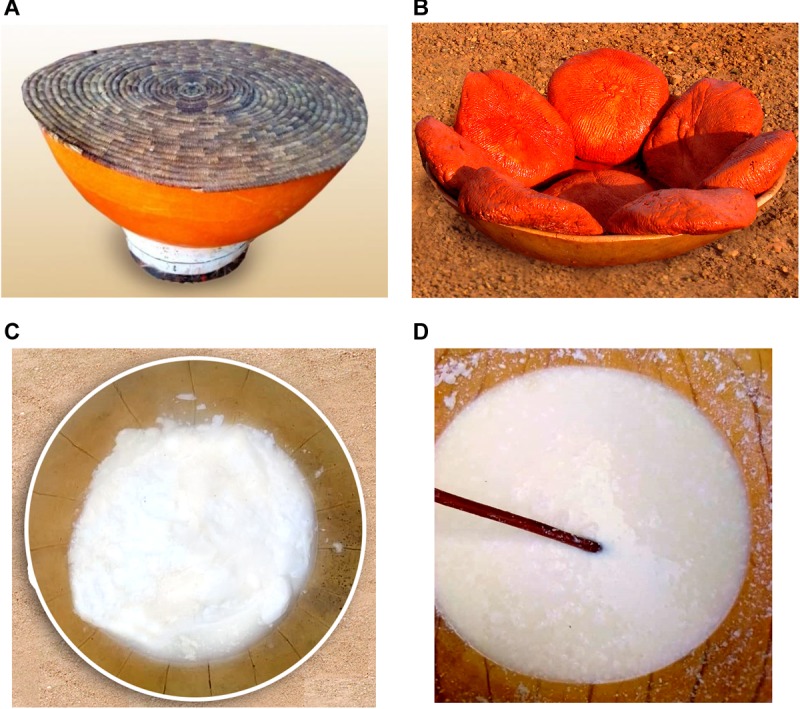
Pictures of the studied dairy products of Benin and Niger. **(A)** The traditional container used for milk fermentation in Niger, **(B)** the pink colored *Wagashi* cheese marketed in Benin, and the naturally fermented milk product of Benin **(C)** and Niger **(D)** are shown here. The colored *Wagashi* cheese is prepared by soaking into a millet leaf brine for the pink color formation.

In most of Sub-Sahara Africa countries, milk production occupies a prominent place due to its economic value, as well as its nutritional, social, and cultural importance (Mattiello et al., 2018). Several projects in the region have aimed at improving the reproductive potential and milk production potential of the local breeds. Republics of Niger and Benin are considered as significant producers of milk in the region with annual cow milk productions of 596,968 and 109,660 tons, respectively (Gagara et al., 2017; Ministry of Agriculture Livestock and Fisheries [MAEP], 2017). Because of its high perishability, the raw milk produced in Benin and Niger is transformed into various derived products by traditional processes. These milk products do not meet regulatory quality requirements and consumer preferences because of unhygienic conditions for milking, uncontrolled processes, and storage practices. Among these, spontaneously fermented milk and *Wagashi* cheese are considered as the major products and most appreciated by the consumers in Benin and Niger (Boko et al., 2016; Dossou et al., 2016; Gagara et al., 2017; Okry et al., 2017). The naturally fermented milk produced in the two countries is obtained from cow milk by incubation of unpasteurized milk for 1 day at room temperature to allow spontaneous fermentation without back-slopping (Figure 1). Fermented milk is commonly used as a dessert or refreshment by people in these countries (Boko et al., 2016). These by-products are characterized by high variability from producer to producer due to uncontrolled spontaneous fermentation (Mattiello et al., 2018). *Wagashi* (also called as *Gassire* in the local Fulfulde language) is a soft fresh cheese produced from cow milk. For its preparation, milk is cleaned from impurities and boiled for about 5 min. Each liter of boiled milk is mixed with about 0.5 L of fresh unheated milk with the extract of *Calotropis procera* leaves (approximately 15 g). The mixture is kept at a warm temperature (60–70°C) until coagulation is achieved. After coagulation, the heating is stopped following the separation of curd and whey. The heated curd is then drained and molded without being pressed and incubated at room temperature (Dossou et al., 2006; Tossou, 2018). *Wagashi* cheese is a source of protein and other essential nutrients, especially for low-income populations and commonly used as a replacement of meat or fish in different dishes (Ministry of Agriculture Livestock and Fisheries [MAEP], 2017). Despite the importance of these dairy products in the diet of the Benin and Niger people, very limited information is currently available on the microbial species associated with these indigenous products. Moreover, understanding the microbial and biochemical changes in these traditional products is the first step toward developing technologies for their safety and quality improvement. Besides bacteria, yeasts frequently occur in dairy products and represent an important component of many cheese varieties and fermented milk products (Fasoli et al., 2015). As a part of starter cultures together with bacteria, yeasts especially relevant in fermented products contribute to the ripening of cheese, enhancing flavor development and accelerating maturation (Garcia et al., 2004; Legras et al., 2018). Thus, special attention is given to the role of yeasts in the complex microbial interactions prevailing in several dairy products and to control fermentation, maturation, and important product characteristics (Jakobsen and Narvhus, 1996). To our knowledge, previous studies conducted on fungal ecology of *Wagashi* cheese and naturally fermented milk from Benin and Niger are limited by cultivation on conventional media (Sessou, 2013). As it is well known that the cultivability of the microbiota is still a limiting factor in understanding the microbial ecology of natural food fermentation, cultivation-independent in-depth metagenomic analysis by amplicon sequencing of the rRNA gene is used to get a realistic view of the microbial community associated with traditionally fermented food products. Most of the next-generation sequencing-based metagenomics studies on fermented milk products described bacterial diversity in the natural milk fermentation (Parker et al., 2018; Shangpliang et al., 2018). There is a need to know the yeast ecology of these milk products to understand their role in the fermentation, maturation, and quality. We aim to use Illumina MiSeq amplicon sequencing of ITS region of rRNA gene amplified from the food metagenome to get a realistic view of yeast community associated with the naturally fermented milk and cheese products of Benin and Niger. The present study is the first cultivation-independent report on yeast community structure present in the naturally fermented milk products from African countries.

## Materials and Methods

### Sampling

Twenty samples of naturally fermented milk were collected from three regions, namely, Niamey, Tillaberi, and Dosso of Niger, and 20 samples of each milk product, namely, *Wagashi* cheese and naturally fermented milk were collected from different geographical regions, namely, Djougou, Parakou, Pehunco, Agouna, and Houeyogbe of Benin. The fermented milk samples were aseptically collected from the producers in Fulani camps whereas *Wagashi* cheese samples were collected from retailers in the markets of targeted areas, transported in an ice-box, and stored in the laboratory at −80°C.

### Metagenomic DNA Extraction

DNA was extracted as per the method described in Keisam et al. (2016) with some minor modifications. Briefly, 2 g of *Wagashi* cheese or 2 mL of fermented milk samples were homogenized with 98 mL 2% sodium citrate buffer in a Stomacher^®^ 400 Circulator (Seward, United Kingdom) at 200 rpm for 2 min. After homogenization, the big debris was allowed to settle down and the clear homogenate was used for DNA extraction; 1.5 mL of the homogenate was transferred to a sterile 2 mL screw-cap tube containing 0.5 g of zirconia/silica beads (0.1 mm) (BioSpec Products, Inc., United States) and four glass beads (2 mm) and centrifuged for 10 min at 18,000 × *g*. To the pellet, 400 μL of TES buffer (50 mM Tris, 1 mM EDTA, 8.7% sucrose, pH 8), 50 KU lysozyme, 25 U mutanolysin, and 20 U lyticase (Sigma-Aldrich, United States) were added and incubated at 37°C for 1 h. After incubation, 25 μL Proteinase-K (25 mg/mL) (Himedia, India) was added to the mixture and further incubated at 65°C for 1 h. This was followed by the addition of 300 μL pre-warmed (65°C) NTS buffer (0.2 M NaCl, 0.1 M Tris, 2% SDS) (Promega, United States) and further incubation at 65°C for 10 min. The DNA was then purified once with phenol and twice with chloroform: isoamyl alcohol mixture (24:1) (Merck, India) each time with centrifugation at 4°C for 15 min at 15,000 × *g*. DNA was precipitated with isopropanol and the DNA pellet dissolved in 50 μL TE buffer (10 mM Tris, 1 mM EDTA) (Sigma-Aldrich, United States). The quality (A_260/280_ and A_260/230_) and quantity of the extracted DNA were measured using spectrophotometer (NanoDrop ND-1000, United States). DNA was stored at −20°C until required.

### Barcoded Illumina MiSeq Sequencing

For in-depth yeast community analyses, barcoded Illumina MiSeq amplicon sequencing targeting the variable internal transcribed spacer (ITS) region was performed using a forward primer (ITS1-F: 5′-CTTGGTCATTTAGAGGAAGTAA-3′) and reverse primer (ITS2: 5′-GCTGCGTTCTTCATCGATGC-3′) (White et al., 1990; Motooka et al., 2017). The 5′ end of the reverse primers was barcoded with a 12-bp error correcting Golay barcodes (Caporaso et al., 2012) to enable sample multiplexing. The complete list of the forward and barcoded reverse primers used in the present study is listed in Supplementary Table S1. Each PCR was performed in a 25 μL reaction volume containing 1X high-fidelity reaction buffer, 1.0 mM MgCl_2_, 0.1 μM forward primer, 0.1 μM reverse primer (IDT, United States), 0.5 U Phusion high-fidelity DNA polymerase (New England Biolabs, Ipswich, MA, United States) and nuclease-free water, with the following conditions: 94°C for 1 min; 94°C for 30 s, 52°C for 30 s and 68°C for 30 s for 35 cycles and 68°C for 7 min. A template-free reaction was used as the control. The PCR products were separated in a 2.0% agarose gel (w/v) and purified using QIAquick gel extraction kit (Qiagen, New Delhi, India) as per the manufacturer’s instructions. The purified DNA was quantified using Qubit dsDNA BR Assay Kit (Invitrogen, United States) in a Qubit 2.0 fluorometer (Invitrogen, Carlsbad, CA, United States) and the individual samples were pooled in equimolar proportions. The final DNA pool was sent to the NGS facility in Xcelris Genomics (Ahmedabad, India) for paired-end Illumina MiSeq sequencing.

### Data Processing

The raw sequence reads obtained was analyzed using QIIME2 version 2019.1^1^ bioinformatics pipeline (Caporaso et al., 2012). Briefly, removal of adaptor sequences, generation of paired-end reads and sample demultiplexing was performed by QIIME2 scripts^2^ in a Linux platform following the procedure used by Romi et al. (2015). The resulting sequences were quality-filtered based on the number of ambiguous bases and Phred quality scores using the default options. Chimera checking and removal of chimeric sequences were performed using the ChimeraSlayer algorithm. The filtered sequences were clustered into operational taxonomic units (OTUs) at 97% identity threshold and picking up a representative sequence in QIIME2 was done by using the furthest-neighbor algorithm. The representative sequences of OTUs were taxonomically annotated using the UNITE fungal ITS database release version 7.1 (Nilsson et al., 2018^3^) using QIIME2 pipeline. The unassigned OTUs were further blasted with UNITE database^4^ and the closest relatives with % of sequence similarity were obtained. Multiple OTUs with closer similarity to a particular yeast species were merged together during the relative abundance estimation. Bacteria-specific and unassigned OTUs were filtered from the final OTU table before performing yeast community statistical analyzes. To establish the phylogenetic relationship of two OTUs of *Saccharomyces cerevisiae* present in the fermented milk samples, the nucleotide sequences (481 bases) of ITS of 26S large subunit ribosomal RNA gene retrieved from the fermented milk metagenome were aligned with the ITS sequences of *S. cerevisiae* cultures of milk and wine origin available in the GenBank by using ClustalW. A neighbor-joining phylogenetic tree based on the Kimura-2 parameter evolutionary distance matrix with 1000 bootstrap replications was constructed from the aligned sequences using MEGA6 software (Tamura et al., 2013). The ITS sequence of *Saccharomyces bayanus* CBS8715 was maintained as outgroup.

### Statistical Analysis

**FIGURE 2:**
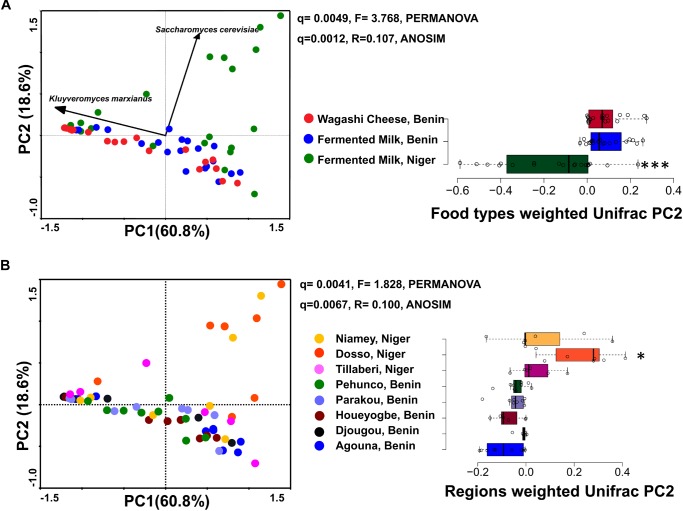
PCA plots show the difference in the yeast community structure among the fermented milk products of Niger and Benin **(A)** and the regions of sample collection **(B)**. The direction of yeast species with significant association is indicated with arrows. PERMANOVA and ANOSIM used to visualize the separation among the groups using Bray–Curtis distance and the significance in difference was expressed as Bonferroni corrected *q-values*. The horizontal box plot shows the principal coordinates of weighted Unifrac that significantly differ among the groups (indicated as ^∗^*q* < 0.05, ^∗∗∗^*q* < 0.001).

The difference in the overall yeast community structure was analyzed by an unsupervised principal component analysis (PCA) clustering using Canoco software v4.52 (Wageningen University, Netherlands). The relative abundance of the filtered yeast species-level OTUs were normalized by log transformation (log xi + 1) before performing the PCA analysis. The principal coordinate shows a significant difference was visualized further with horizontal boxplot using BoxPlotR^5^. The significant difference in the yeast community structure of different food types or regions was calculated by PERMANOVA test with 9999 permutations using Bray–Curtis distances in PAST v3.08 (Hammer et al., 2001). ANOSIM also calculated to support the significant difference. The main diversity indices used for ecological application, namely, Chao-1, Shannon, Simpson, Evenness, Dominance, Fisher alpha, Berger–Parker indices (Morris et al., 2014) were analyzed to show the difference in yeast diversity between the food types and regions. Whittaker index was calculated to show the beta diversity. The diversity indices were calculated using the species level OTUs using PAST. ANOVA was performed to show the statistical difference in yeast diversity. Any significant difference in the abundance of specific taxa or diversity between the food groups or the region was calculated as “Bonferroni” corrected *p-value* using R (v3.1.3). The relative abundance (%) of yeast taxa was used for generating the bar chart (Figure 2A). We tested the yeast taxa that differently abundant across the sample groups (country, food type, and region of sample collection) with ANCOM analysis (Mandal et al., 2015) by using QIIME2. The OTU table file along with sample metadata (country, food type, and region of sample collection) was used for ANCOM visualization. The statistical significance of the difference in the abundance of yeast taxa between the groups (food types or regions) was also obtained by Wilcoxon test with Bonferroni corrected *p-value* (*q*-value) using “svDialogs” in R package (v3.1.3). The yeast taxa showed significant differential abundance between the food types were visualized with boxplots using BoxPlotR.

### Sensory Analysis

The texture of the traditionally fermented milk products of Niger and Benin were analyzed by consistency grading as per the International Dairy Federation (IDF) (Karagül-Yüceer and Drake, 2007). Twenty samples each of marketed fermented milk from Benin and Niger were analyzed for the consistency grades of setting, lumps or flakes, dripping, gritty, sticky, too thick, too fluid, ropy/stringy, dried, brittle, and gelatinous nature.

### Data Accession

The yeast ITS sequences generated in the present study was deposited in NCBI SRA with the accession number PRJNA506750 (Supplementary Data Sheets S1, S2).

## Results

The yeast communities of the naturally fermented milk from Niger and Benin and the *Wagashi* cheese from Benin were analyzed for the first time by MiSeq amplicon sequencing of fungal ITS region. This in-depth analysis resulted in a total of 13,51,858 quality-filtered sequences of 200–550 bp read length with an average reads of 22,530 ± 8464 per sample. The taxonomic assignment of the sequences at 97% identity in UNITE database resulted with 1548 yeast OTUs. The yeast community structure of the fermented milk products was compared based on the yeast species-level OTUs relative abundance profiles by a PCA using Bray–Curtis distance metrics. The yeast community structure in fermented milk products of Niger and Benin significantly differed (*q* = 0.001, *F* = 3.4, PERMANOVA) (Figure 2A). The principal coordinate-2 that showed a distinct separation between the two countries was visualized with a boxplot. Pair-wise ANOVA comparison showed that the yeast community structure of fermented milk of Niger differed significantly with fermented milk of Benin (*q* = 0.015, Bonferroni corrected) and *Wagashi* cheese of Benin (*q* = 0.0069, Bonferroni corrected). Further analysis to see the yeast community difference across the sample collection regions also showed a significant difference (*q* = 0.0041, PERMANOVA) (Figure 2B). ANOVA pair-wise comparison showed a significant difference in the yeast community of fermented milk samples collected between the Dosso region of Niger, and Houeyogbe (*q* = 0.042, Bonferroni corrected) and Parakou (*q* = 0.042, Bonferroni corrected) regions of Benin. We also noticed a significant difference in the diversity indices between the fermented milk products of Niger and Benin (Table 1). The fermented milk of Benin had a high yeast species richness (Chao-1) and high yeast diversity (Shannon index) than the other two milk products (*q* < 0.05, ANOVA). The samples collected from Pehunco and Djougou regions of Benin had low yeast richness and significantly differed with the samples from Niamey and Tillaberi regions of Niger (*q* < 0.05, Bonferroni corrected). Moreover, beta diversity analysis resulted with Whittaker index value of 20.42. Our results supported the association of different yeast community structure in the fermented milk products of Benin and Niger.

**Table 1:** Yeast diversity in fermented milk products collected from different regions of Benin and Niger.

	Chao-1	Shannon_H	Simpson_1-D	Evenness_e∧H/S	Dominance_D	Fisher_alpha	Berger-Parker
**Food**							
Fermented milk, Niger	80.75 ± 21.98	1.45 ± 0.20	0.53 ± 0.06	0.13 ± 0.04	0.47 ± 0.06	13.29 ± 2.66	0.59 ± 0.06
Fermented milk, Benin	110.30 ± 42.90	2.01 ± 0.21	0.69 ± 0.05	0.37 ± 0.06	0.31 ± 0.05	15.16 ± 8.92	0.45 ± 0.05
Wagashi cheese, Benin	26.11 ± 6.01	1.29 ± 0.20	0.48 ± 0.08	0.39 ± 0.08	0.52 ± 0.08	10.33 ± 3.17	0.61 ± 0.07
**Region**							
Agouna, Benin	91.80 ± 48.76	1.84 ± 0.37	0.60 ± 0.11	0.36 ± 0.11	0.40 ± 0.11	13.42 ± 6.14	0.49 ± 0.11
Djougou, Benin	35.33 ± 17.09	0.91 ± 0.31	0.35 ± 0.13	0.25 ± 0.11	0.65 ± 0.13	5.18 ± 1.53	0.75 ± 0.10
Houeyogbe, Benin	98.86 ± 72.12	1.91 ± 0.39	0.68 ± 0.11	0.48 ± 0.14	0.32 ± 0.11	9.56 ± 4.28	0.42 ± 0.09
Parakou, Benin	95.38 ± 64.78	1.76 ± 0.38	0.58 ± 0.11	0.26 ± 0.08	0.42 ± 0.11	28.12 ± 21.09	0.56 ± 0.10
Pehunco, Benin	15.11 ± 2.39	1.57 ± 0.16	0.63 ± 0.06	0.47 ± 0.10	0.37 ± 0.06	5.47 ± 1.20	0.52 ± 0.06
Dosso, Niger	50.14 ± 24.55	1.41 ± 0.26	0.55 ± 0.09	0.21 ± 0.09	0.45 ± 0.09	9.38 ± 2.78	0.58 ± 0.09
Niamey, Niger	102.00 ± 49.45	1.69 ± 0.37	0.57 ± 0.10	0.10 ± 0.02	0.43 ± 0.10	13.56 ± 3.85	0.57 ± 0.10
Tillaberi, Niger	91.67 ± 30.51	1.21 ± 0.36	0.46 ± 0.11	0.06 ± 0.01	0.54 ± 0.11	17.52 ± 6.47	0.64 ± 0.11

*Data presented as mean ± standard error mean.*

**FIGURE 3:**
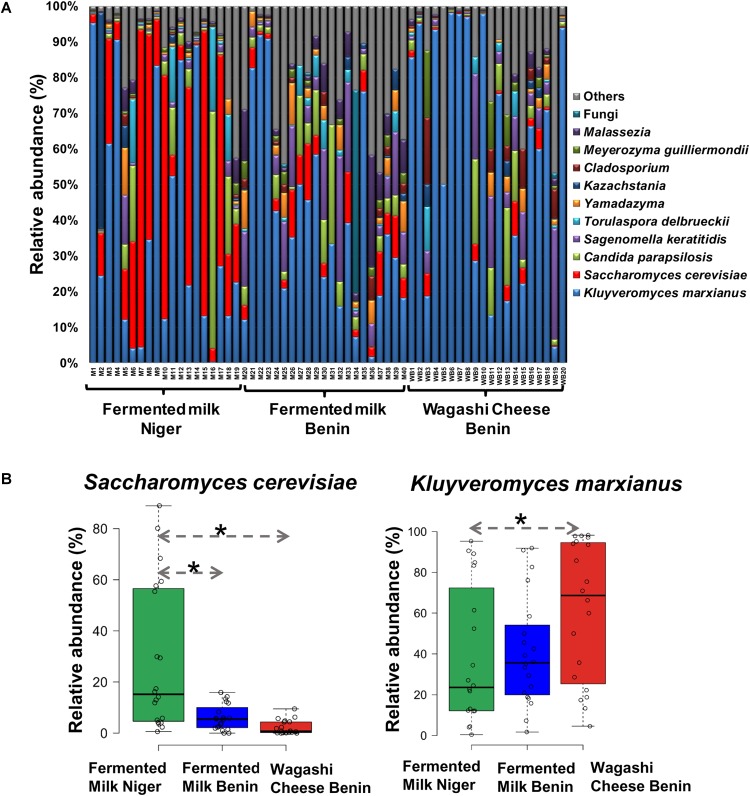
**(A)** Yeast taxon plot shows the relative abundance of predominant yeast present in the naturally fermented milk products of Benin and Niger. Each column represents the relative abundance (%) of yeast taxa investigated by using Illumina MiSeq amplicon sequencing of ITS region. OTUs with similar species-level identity at 97% similarity in the UNITE database are merged here. Taxa with less than 1% mean relative abundance across the samples studied are combined and shown as others. **(B)** Boxplot shows the differential abundance of *Saccharomyces cerevisiae* and *Kluyveromyces marxianus* among the fermented milk products of Niger and Benin. The significance in difference was calculated by Wilcoxon test with Bonferroni correction and indicated as ^∗^*q* < 0.05.

**FIGURE 4:**
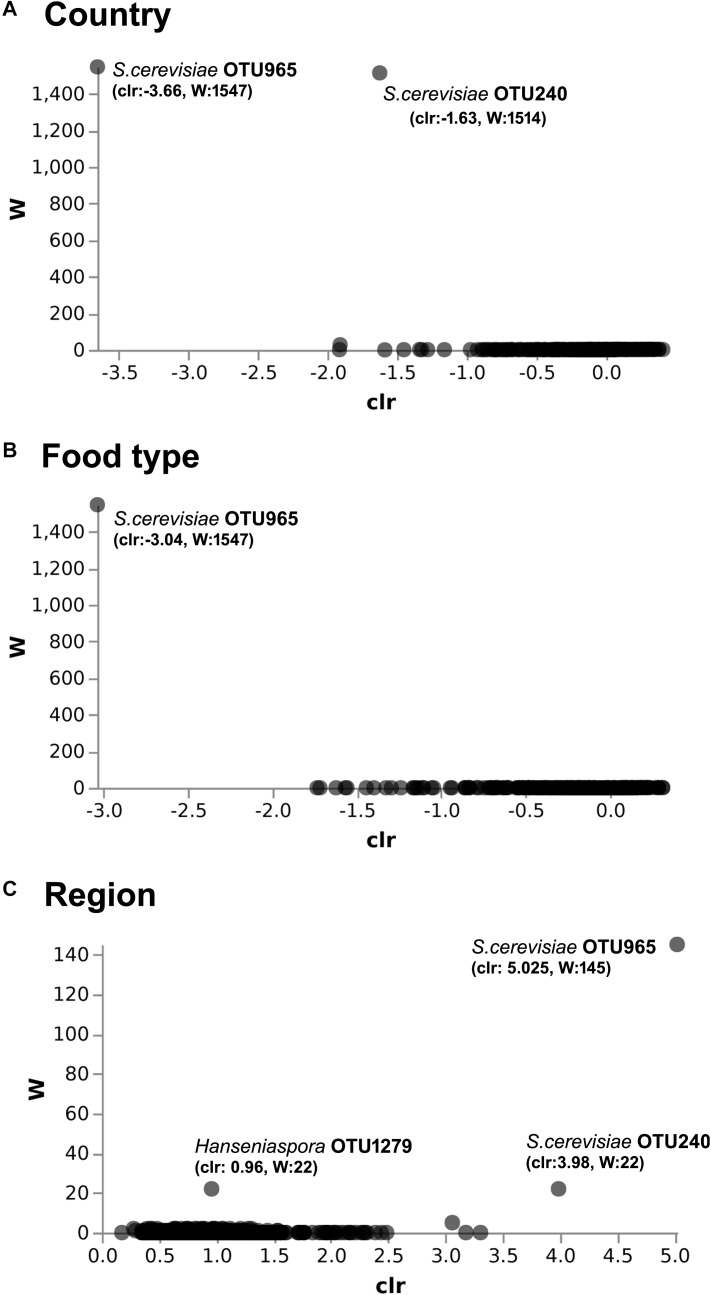
ANCOM differential abundance testing shows the yeast taxa significantly differ across the **(A)** country (Benin and Niger) and **(B)** food type (fermented milk and cheese). **(C)** No significant difference observed between the regions of sample collection. QIIME2 version 2019.1 was used for ANCOM analysis. The percentile abundances of features by the group of significantly differing yeast taxa are shown in Table 2.

**FIGURE 5:**
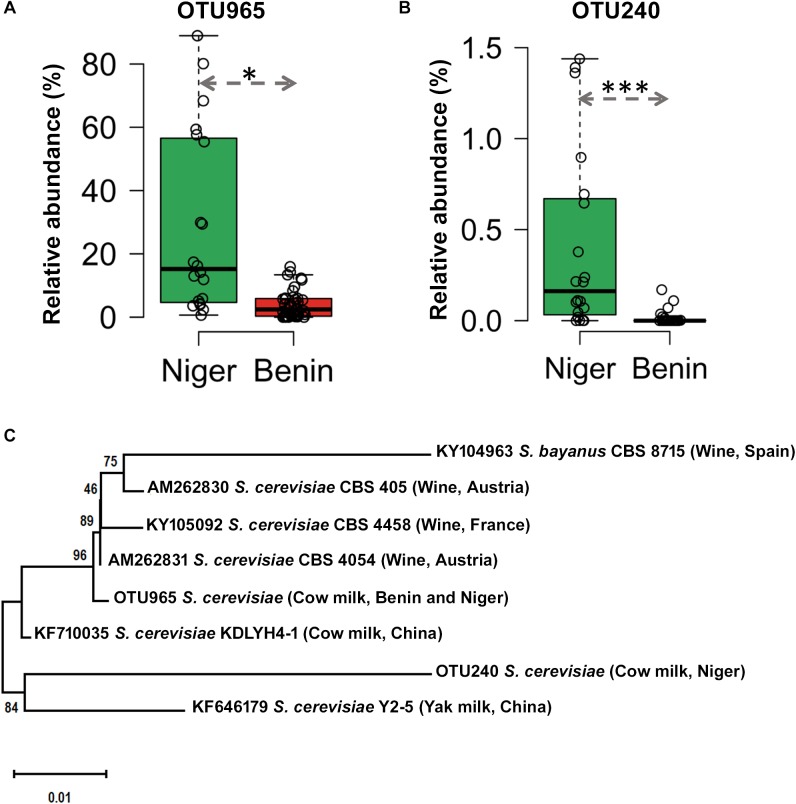
Difference in the occurrence and phylogenetic affiliation of two OTUs of *Saccharomyces cerevisiae* retrieved from the metagenome of fermented milk products of Niger and Benin. **(A,B)** Box plots shows the difference in the occurrence of OTU965 and OTU240 in the fermented milk products of Niger and Benin. The significance in difference was calculated by Wilcoxon test with Bonferroni correction and indicated as ^∗^*q* < 0.05, ^∗∗∗^*q* < 0.001. **(C)** The neighbor-joining phylogenetic tree generated based on the sequences of the internal transcribed spacer (ITS) region of 26S large subunit ribosomal RNA gene of the two OTUs of *S. cerevisiae* in comparison with the ITS sequence of earlier characterized cultures of *S. cerevisiae* shows its close relatives of wine and milk origins. *Saccharomyces bayanus* CBS8715 was maintained as an out-group. The evolutionary distances were computed using the Kimura two-parameter method and the scale indicates the units of the number of base substitutions per site.

The predominant yeast taxa associated with the fermented milk products of Benin and Niger are shown with a relative abundance graph (Figure 3A). *Kluyveromyces marxianus* (39.42%), *S. cerevisiae* (17.28%)*, Candida parapsilosis* (7.23%), and *Sagenomella keratitidis* (5.62%) were predominant in the naturally fermented milk of Benin and Niger. Regarding the cheese samples, *K. marxianus* (61.13%), *S. keratitidis* (6.44%)*, C. parapsilosis* (4.93%), and *S. cerevisiae* (4.65%) were prevalent. Further analysis showed that *S. cerevisiae* as the key differentiating taxa (*q* < 0.05, Wilcoxon test, Bonferroni corrected) with a high presence in the fermented milk products of Niger (Figure 3B). We used ANCOM differential abundance analysis using QIIME2 to visualize the yeast taxa that significantly differed among the samples by group (country, food type, and the region of sample collection). ANCOM visualization (Figure 4) showed that two OTUs of *S. cerevisiae* (OTU965 and OTU240) significantly differed between Benin and Niger. The *W-value* for the two OTUs is much higher (>1500) than other yeast taxa. Whereas OTU240 was exclusively present in the fermented milk of Niger (Figure 5A,B). Table 2 shows the ANCOM percentile abundance of features by the group for the significantly differing yeast taxa. We did not see any yeast taxa that significantly differed between the regions of sample collection within two countries. The neighbor-joining phylogenetic tree (Figure 5C) generated based on the nucleotide sequence similarity of ITS region of 26S rRNA gene showed the closest relative of the two OTUs of *S. cerevisiae* present in the fermented milk products. OTU965 showed a closer similarity with the *S. cerevisiae* strains of wine origin. Whereas, OTU240 showed a divergent with *S. cerevisiae* strains of milk origin. We observed a high presence of *K. marxianus* in the *Wagashi* cheese of Benin (Figure 3C). Also noticed a denser texture in the *K. marxianus* predominated fermented milk of Benin, whereas lighter texture in the *S. cerevisiae* predominated fermented milk of Niger. The other way around, the low presence of *S. cerevisiae* associated with the denser structure and low presence of *K. marxianus* associated with the lighter texture. Consistency analysis by IDF grading (Table 3) showed that most of the samples (16/20) from Benin showed thick texture with lumps and flakes. Whereas, the samples of Niger (18/20) showed with too fluid with dripping texture.

**Table 2:** ANCOM differential abundance testing resulted significantly differing yeast taxa and their percentile abundance of samples by group.

Group (country)	Benin					Niger				
Percentile	0	25	50	75	100	0	25	50	75	100
*Saccharomyces cerevisiae* OTU965	1	2	5	10	5169	5	104	186	2746	24,551
*Saccharomyces cerevisiae* OTU240	1	1	1	1	57	1	2	6	39	369

**Group (food type)**	**Cheese**					**Fermented milk**				
	
Percentile	0	25	50	75	100	0	25	50	75	100
*Saccharomyces cerevisiae* OTU965	1	2	3	5	1082	1	6.5	100	1681	24,551
Group (region)	No statistical significance									

**Table 3:** Difference in the consistency of the naturally fermented milk products marketed in Benin and Niger.

Source	Number of samples	Consistency grading (IDF, 1997)										
		Setting	Lumps or flakes	Dripping	Gritty	Sticky	Too thick	Too fluid	Ropy/ stringy	Dried	Brittle	Gelatinous
Fermented milk of Benin	20	16	16	4	0	0	16	4	0	0	0	16
Fermented milk of Niger	20	2	2	18	0	0	2	18	0	0	0	2

*The texture judging criteria as consistency grading were followed from the guidelines of International Dairy Federation (IDF) (Karagül-Yüceer and Drake, 2007).*

## Discussion

Yeast and lactic acid bacteria together contribute to the characteristic taste, texture, and flavor of naturally fermented milk products. The difference in their composition contributes to the differences in chemical composition and led to a variety of milk products. The present study investigated the yeast ecology of naturally fermented milk products of Benin and Niger and showed that the yeast composition differed between the countries. The difference in the yeast composition in the studied products may be linked to the difference in indigenous production processes, geographical location, different climatic conditions, and grassland ecosystems of the two countries. Two yeast species, *K. marxianus* and *S. cerevisiae*, dominated the fermented milk products of Benin and Niger. Earlier studies observed *S. cerevisiae*, *K. marxianus*, *Kluyveromyces lactis*, *Debaromyces hansenii*, and *Yarrowia lipolytica* (Rai and Jeyaram, 2017; Hittinger et al., 2018) as the main yeast species present in the similar type of naturally fermented milk products. Similar to the *Wagashi* cheese of Benin, *K. marxianus* dominantly present in several naturally fermented milk products, namely, Indian hard cheese prepared from yak milk “churpi” (Rai et al., 2016) and traditional Mongolian fermented mare’s milk “airag” (Watanabe et al., 2008). The dominance of *S. cerevisiae* in natural milk fermentation also reported from African milk products, “nyarmie” of Ghana (Obodai and Dodd, 2006) and “amabere amaruranu” of Kenya (Nyambane et al., 2014). *Pichia kudriavzevii* along with *S. cerevisiae* dominated in a West African fermented yogurt-like milk product – “nunu” (Owusu-Kwarteng et al., 2017). Among the other yeast species *C. parapsilosis*, *S. keratitidis*, *Torulaspora delbrueckii*, *Yamadazyma*, *Kazachstania*, *Cladosporium*, *Meyerozyma guilliermondii*, and *Malassezia* were also relatively abundant in the studied milk products. Whereas earlier studies showed the presence of *Galactomyces geotrichum*, *Issatchenkia orientalis*, *Pichia mandshurica*, *Pichia fermentans*, *P. kudriavzevii*, *Candida lusitaniae*, *Candida rugosa*, *Candida tropicalis* in several dairy products (Shangpliang et al., 2018). In addition, the predominance of *Galctomyces* spp. (Murugesan et al., 2018) and *Debaryomyces* spp. (Dos Santos et al., 2017) reported in several cheese varieties. The presence of diverse yeast species and their compositional difference is linked with the unique sensory properties of the different naturally fermented milk products.

The dominance of *K. marxianus* in the investigated milk products is a good trait as this food-grade thermotolerant yeast produces a wide array of volatile molecules and contributes to the flavor development of different fermented beverages (Morrissey et al., 2015). It is a lactose-fermenting yeast conventionally recognized as *Candida kefyr* and phylogenetically related to *S. cerevisiae*, which possesses biotechnological and probiotic potential including the ability to survive in the gastric and intestinal environments (Gethins et al., 2016). Several studies have recognized *K. marxianus* as an emerging probiotic, fermentation starter with generally regarded as safe (GRAS) status. *K. marxianus* is used as a starter culture along with *Lactobacillus delbrueckii* subsp. *bulgaricus* and *Streptococcus thermophilus* to enhance the flavor (Zhang et al., 2017), taste (Fasoli et al., 2016), and denser texture (Gethins et al., 2016). The denser texture observed in the fermented milk of Benin with high abundance of *K. marxianus* or low abundance of *S. cerevisiae*, and lighter texture in the fermented milk of Niger with high abundance of *S. cerevisiae* or low abundance of *K. marxianus* is worthy to study further on developing a product with a difference in texture. Besides *K. marxianus*, non-lactose-fermenting *S. cerevisiae* is also recognized as a potential starter culture for fermented milk production with improved qualities (FAO/WHO/CODEX ALIMENTARIUS, 2018). Further studies on selective isolation of the strain of *S. cerevisiae* OTU240 exclusively present in the fermented milk of Niger and characterizing in the angle of its association with milk products is worthwhile. The dominance of *K. marxianus* and *S. cerevisiae* could significantly contribute to the organoleptic profile of these dairy products by producing CO_2_ as well as aroma compounds (Aponte et al., 2010), thus provide an advantage of improving texture and flavor. Further studies on chemical profiling by mass spectrometry will give an insight into the role of yeast in the biotransformation of milk fermentation.

The identified yeast species may contribute significantly to many desirable effects in the fermented milk products in one hand and potential pathogens in the other hand. The presence of *S. keratitidis* and *C. parapsilosis* in the investigated products may be of risk for the consumers. This is the first report on the predominance of *S. keratitidis* in the naturally fermented milk products of Africa. *S. keratitidis* is a potential agent causing keratitis, inflammation of cornea and eyes. Moreover, *S. keratitidis* found to be an emerging agent of nail infection (Tsang, 2017). Systemic fungal infection by *Sagenomella* lead to Juvenile idiopathic arthritis has been also reported (Ried and Fakler, 2018). Indeed, *C. parapsilosis* is recognized as an emerging major human pathogen that causes invasive candidal disease (Trofa et al., 2008). Recently, *C. parapsilosis* has been recognized as the most frequent non-albicans Candida (NAC) species of invasive candidiasis in neonates (Garzillo et al., 2017). However, several reports observed the presence of *C. parapsilosis* in traditional food fermentation (Chaves-López et al., 2014). Similarly, *M. guilliermondiii* regarded as an emerging infectious yeast of the NAC observed in the studied milk products also predominately involved in the natural bamboo shoot fermentation (Romi et al., 2014). The presence of potential yeast pathogens in the uncontrolled natural milk fermentation and its impact on health particularly on children to be studied further to confirm its safety. As *S. keratitidis* infect the nails, unhygienic milking and further spread through marketing of fermented milk products require an epidemiological study. Further studies on understanding the biochemical changes by different yeast species in a controlled milk fermentation will improve our understanding on the role of yeast in milk fermentation.

## Conclusion

This study provides a baseline understanding of the yeast ecology of traditional fermented milk products of the two African countries Benin and Niger. The results showed that *K. marxianus* and *S. cerevisiae* were the prevalent yeast species which may have an important role during natural milk fermentation and *Wagashi* cheese production. The composition difference of these two yeast species might contribute to the differences in the physical and chemical character of the studied products. Further studies are necessary to selectively isolate the predominant indigenous yeasts *K. marxianus* and *S. cerevisiae* and screen them for desirable fermentative and functional properties with aim of developing a controlled milk fermentation for producing safe and quality milk products. The presence of potential pathogenic yeasts *S. keratitidis* and *C. parapsilosis* in these traditional foods is to be further investigated to assess safety. The study offers tremendous opportunities to predict and control the growth and survival of desirable or undesirable microorganisms in the studied products.

## Data Availability

All datasets generated for this study are included in the manuscript and/or the Supplementary Files.

## Author Contributions

PS, MG, and KJ conceived and designed the research. PS and SK acquired the data, interpreted the results, and wrote the manuscript. NT assisted with UNITE database analysis. KJ and SF critically revised the manuscript. All authors read and approved the final manuscript.

## Conflict of Interest Statement

The authors declare that the research was conducted in the absence of any commercial or financial relationships that could be construed as a potential conflict of interest.

## Footnotes

1 https://docs.qiime2.org/2019.1/

2 https://docs.qiime2.org/2019.1/tutorials/

3 http://unite.ut.ee

4 https://unite.ut.ee/analysis.php

5 http://shiny.chemgrid.org/boxplotr/
