# Supplementary material for: High-Throughput Illumina MiSeq Amplicon Sequencing of Yeast Communities Associated With Indigenous Dairy Products From Republics of Benin and Niger
Source: Front Microbiol. 2019 Apr 3;10:594. doi: 10.3389/fmicb.2019.00594 (PMC6456676; doi:10.3389/fmicb.2019.00594)
Supplement: Supplementary file 1 [file Table_1.docx]

**Supplementary Information**

**Title:** High-throughput Illumina MiSeq amplicon sequencing of yeast communities associated with indigenous dairy products from Republics of Benin and Niger

**Authors:** Philippe Sessou, Santosh Keisam, Ngangyola Tuikhar, Mariama Gagara, Souaïbou Farougou, Kumaraswamy Jeyaram

**Supplementary Table S1:** Barcoded primers used for amplification of internal transcribed spacer region (ITS) of fungal rRNA genes, and multiplexing the samples for MiSeq amplicon sequencing

|  | Oligo Name | Sequence (5’-3’) |
| --- | --- | --- |
| Forward | ITS1f | CTTGGTCATTTAGAGGAAGTAA |
| Reverse | MID1-ITS2 | **TCCCTTGTCTCC**GCTGCGTTCTTCATCGATGC |
|  | MID2-ITS2 | **ACGAGACTGATT**GCTGCGTTCTTCATCGATGC |
|  | MID3-ITS2 | **GCTGTACGGATT**GCTGCGTTCTTCATCGATGC |
|  | MID4-ITS2 | **ATCACCAGGTGT**GCTGCGTTCTTCATCGATGC |
|  | MID5-ITS2 | **TGGTCAACGATA**GCTGCGTTCTTCATCGATGC |
|  | MID6-ITS2 | **ATCGCACAGTAA**GCTGCGTTCTTCATCGATGC |
|  | MID7-ITS2 | **GTCGTGTAGCCT**GCTGCGTTCTTCATCGATGC |
|  | MID8-ITS2 | **AGCGGAGGTTAG**GCTGCGTTCTTCATCGATGC |
|  | MID9-ITS2 | **ATCCTTTGGTTC**GCTGCGTTCTTCATCGATGC |
|  | MID10-ITS2 | **TACAGCGCATAC**GCTGCGTTCTTCATCGATGC |
|  | MID11-ITS2 | **ACCGGTATGTAC**GCTGCGTTCTTCATCGATGC |
|  | MID12-ITS2 | **AATTGTGTCGGA**GCTGCGTTCTTCATCGATGC |
|  | MID13-ITS2 | **TGCATACACTGG**GCTGCGTTCTTCATCGATGC |
|  | MID14-ITS2 | **AGTCGAACGAGG**GCTGCGTTCTTCATCGATGC |
|  | MID15-ITS2 | **ACCAGTGACTCA**GCTGCGTTCTTCATCGATGC |
|  | MID16-ITS2 | **GAATACCAAGTC**GCTGCGTTCTTCATCGATGC |
|  | MID17-ITS2 | **GTAGATCGTGTA**GCTGCGTTCTTCATCGATGC |
|  | MID18-ITS2 | **TAACGTGTGTGC**GCTGCGTTCTTCATCGATGC |
|  | MID19-ITS2 | **CATTATGGCGTG**GCTGCGTTCTTCATCGATGC |
|  | MID20-ITS2 | **CCAATACGCCTG**GCTGCGTTCTTCATCGATGC |
|  | MID21-ITS2 | **GATCTGCGATCC**GCTGCGTTCTTCATCGATGC |
|  | MID22-ITS2 | **CAGCTCATCAGC**GCTGCGTTCTTCATCGATGC |
|  | MID23-ITS2 | **CAAACAACAGCT**GCTGCGTTCTTCATCGATGC |
|  | MID24-ITS2 | **GCAACACCATCC**GCTGCGTTCTTCATCGATGC |
|  | MID25-ITS2 | **GCGATATATCGC**GCTGCGTTCTTCATCGATGC |
|  | MID26-ITS2 | **CGAGCAATCCTA**GCTGCGTTCTTCATCGATGC |
|  | MID27-ITS2 | **AGTCGTGCACAT**GCTGCGTTCTTCATCGATGC |
|  | MID28-ITS2 | **GTATCTGCGCGT**GCTGCGTTCTTCATCGATGC |
|  | MID29-ITS2 | **CGAGGGAAAGTC**GCTGCGTTCTTCATCGATGC |
|  | MID30-ITS2 | **CAAATTCGGGAT**GCTGCGTTCTTCATCGATGC |
|  | MID31-ITS2 | **AGATTGACCAAC**GCTGCGTTCTTCATCGATGC |
|  | MID32-ITS2 | **AGTTACGAGCTA**GCTGCGTTCTTCATCGATGC |
|  | MID33-ITS2 | **GCATATGCACTG**GCTGCGTTCTTCATCGATGC |
|  | MID34-ITS2 | **CAACTCCCGTGA**GCTGCGTTCTTCATCGATGC |
|  | MID35-ITS2 | **TTGCGTTAGCAG**GCTGCGTTCTTCATCGATGC |
|  | MID36-ITS2 | **TACGAGCCCTAA**GCTGCGTTCTTCATCGATGC |
|  | MID37-ITS2 | **CACTACGCTAGA**GCTGCGTTCTTCATCGATGC |
|  | MID38-ITS2 | **TGCAGTCCTCGA**GCTGCGTTCTTCATCGATGC |
|  | MID39-ITS2 | **ACCATAGCTCCG**GCTGCGTTCTTCATCGATGC |
|  | MID40-ITS2 | **TCGACATCTCTT**GCTGCGTTCTTCATCGATGC |
|  | MID41-ITS2 | **GAACACTTTGGA**GCTGCGTTCTTCATCGATGC |
|  | MID42-ITS2 | **GAGCCATCTGTA**GCTGCGTTCTTCATCGATGC |
|  | MID43-ITS2 | **TTGGGTACACGT**GCTGCGTTCTTCATCGATGC |
|  | MID44-ITS2 | **AAGGCGCTCCTT**GCTGCGTTCTTCATCGATGC |
|  | MID45-ITS2 | **TAATACGGATCG**GCTGCGTTCTTCATCGATGC |
|  | MID46-ITS2 | **TCGGAATTAGAC**GCTGCGTTCTTCATCGATGC |
|  | MID47-ITS2 | **TGTGAATTCGGA**GCTGCGTTCTTCATCGATGC |
|  | MID48-ITS2 | **CATTCGTGGCGT**GCTGCGTTCTTCATCGATGC |
|  | MID49-ITS2 | **TACTACGTGGCC**GCTGCGTTCTTCATCGATGC |
|  | MID50-ITS2 | **GGCCAGTTCCTA**GCTGCGTTCTTCATCGATGC |
|  | MID51-ITS2 | **GATGTTCGCTAG**GCTGCGTTCTTCATCGATGC |
|  | MID52-ITS2 | **CTATCTCCTGTC**GCTGCGTTCTTCATCGATGC |
|  | MID53-ITS2 | **ACTCACAGGAAT**GCTGCGTTCTTCATCGATGC |
|  | MID54-ITS2 | **ATGATGAGCCTC**GCTGCGTTCTTCATCGATGC |
|  | MID55-ITS2 | **GTCGACAGAGGA**GCTGCGTTCTTCATCGATGC |
|  | MID56-ITS2 | **TGTCGCAAATAG**GCTGCGTTCTTCATCGATGC |
|  | MID57-ITS2 | **CATCCCTCTACT**GCTGCGTTCTTCATCGATGC |
|  | MID58-ITS2 | **TATACCGCTGCG**GCTGCGTTCTTCATCGATGC |
|  | MID59-ITS2 | **AGTTGAGGCATT**GCTGCGTTCTTCATCGATGC |
|  | MID-60ITS2 | **ACAATAGACACC**GCTGCGTTCTTCATCGATGC |
