# Supplementary material for: High-Throughput Illumina MiSeq Amplicon Sequencing of Yeast Communities Associated With Indigenous Dairy Products From Republics of Benin and Niger
Source: Front Microbiol. 2019 Apr 3;10:594. doi: 10.3389/fmicb.2019.00594 (PMC6456676; doi:10.3389/fmicb.2019.00594)
Supplement: Supplementary file 2 [file Data_Sheet_1.PDF]

Table 2 : Biosample accessions

Accession	Sample Name	SPUID Organism	Tax ID	BioProject
SAMN10473383	M1	milk metagenome	1616037	PRJNA506750
SAMN10473384	M2	milk metagenome	1616037	PRJNA506750
SAMN10473385	M3	milk metagenome	1616037	PRJNA506750
SAMN10473386	M4	milk metagenome	1616037	PRJNA506750
SAMN10473387	M5	milk metagenome	1616037	PRJNA506750
SAMN10473388	M6	milk metagenome	1616037	PRJNA506750
SAMN10473389	M7	milk metagenome	1616037	PRJNA506750
SAMN10473390	M8	milk metagenome	1616037	PRJNA506750
SAMN10473391	M9	milk metagenome	1616037	PRJNA506750
SAMN10473392	M10	milk metagenome	1616037	PRJNA506750
SAMN10473393	M11	milk metagenome	1616037	PRJNA506750
SAMN10473394	M12	milk metagenome	1616037	PRJNA506750
SAMN10473395	M13	milk metagenome	1616037	PRJNA506750
SAMN10473396	M14	milk metagenome	1616037	PRJNA506750
SAMN10473397	M15	milk metagenome	1616037	PRJNA506750
SAMN10473398	M16	milk metagenome	1616037	PRJNA506750
SAMN10473399	M17	milk metagenome	1616037	PRJNA506750
SAMN10473400	M18	milk metagenome	1616037	PRJNA506750
SAMN10473401	M19	milk metagenome	1616037	PRJNA506750
SAMN10473402	M20	milk metagenome	1616037	PRJNA506750
SAMN10473403	M21	milk metagenome	1616037	PRJNA506750
SAMN10473404	M22	milk metagenome	1616037	PRJNA506750
SAMN10473405	M23	milk metagenome	1616037	PRJNA506750
SAMN10473406	M24	milk metagenome	1616037	PRJNA506750
SAMN10473407	M25	milk metagenome	1616037	PRJNA506750
SAMN10473408	M26	milk metagenome	1616037	PRJNA506750
SAMN10473409	M27	milk metagenome	1616037	PRJNA506750
SAMN10473410	M28	milk metagenome	1616037	PRJNA506750
SAMN10473411	M29	milk metagenome	1616037	PRJNA506750
SAMN10473412	M30	milk metagenome	1616037	PRJNA506750
SAMN10473413	M31	milk metagenome	1616037	PRJNA506750
SAMN10473414	M32	milk metagenome	1616037	PRJNA506750
SAMN10473415	M33	milk metagenome	1616037	PRJNA506750
SAMN10473416	M34	milk metagenome	1616037	PRJNA506750
SAMN10473417	M35	milk metagenome	1616037	PRJNA506750
SAMN10473418	M36	milk metagenome	1616037	PRJNA506750
SAMN10473419	M37	milk metagenome	1616037	PRJNA506750
SAMN10473420	M38	milk metagenome	1616037	PRJNA506750
SAMN10473421	M39	milk metagenome	1616037	PRJNA506750
SAMN10473422	M40	milk metagenome	1616037	PRJNA506750
SAMN10473423	WB1	milk metagenome	1616037	PRJNA506750
SAMN10473424	WB2	milk metagenome	1616037	PRJNA506750
SAMN10473425	WB3	milk metagenome	1616037	PRJNA506750

SAMN10473426	WB4	milk metagenome	1616037	PRJNA506750
SAMN10473427	WB5	milk metagenome	1616037	PRJNA506750
SAMN10473428	WB6	milk metagenome	1616037	PRJNA506750
SAMN10473429	WB7	milk metagenome	1616037	PRJNA506750
SAMN10473430	WB8	milk metagenome	1616037	PRJNA506750
SAMN10473431	WB9	milk metagenome	1616037	PRJNA506750
SAMN10473432	WB10	milk metagenome	1616037	PRJNA506750
SAMN10473433	WB11	milk metagenome	1616037	PRJNA506750
SAMN10473434	WB12	milk metagenome	1616037	PRJNA506750
SAMN10473435	WB13	milk metagenome	1616037	PRJNA506750
SAMN10473436	WB14	milk metagenome	1616037	PRJNA506750
SAMN10473437	WB15	milk metagenome	1616037	PRJNA506750
SAMN10473438	WB16	milk metagenome	1616037	PRJNA506750
SAMN10473439	WB17	milk metagenome	1616037	PRJNA506750
SAMN10473440	WB18	milk metagenome	1616037	PRJNA506750
SAMN10473441	WB19	milk metagenome	1616037	PRJNA506750
SAMN10473442	WB20	milk metagenome	1616037	PRJNA506750
