# Supplementary material for: High-Throughput Illumina MiSeq Amplicon Sequencing of Yeast Communities Associated With Indigenous Dairy Products From Republics of Benin and Niger
Source: Front Microbiol. 2019 Apr 3;10:594. doi: 10.3389/fmicb.2019.00594 (PMC6456676; doi:10.3389/fmicb.2019.00594)
Supplement: Supplementary file 3 [file Data_Sheet_2.PDF]

Table 3 : Links to access Biosample records

10473383	:	https://www.ncbi.nlm.nih.gov/sra/10473383
10473384	:	https://www.ncbi.nlm.nih.gov/sra/10473384
10473385	:	https://www.ncbi.nlm.nih.gov/sra/10473385
10473386	:	https://www.ncbi.nlm.nih.gov/sra/10473386
10473387	:	https://www.ncbi.nlm.nih.gov/sra/10473387
10473388	:	https://www.ncbi.nlm.nih.gov/sra/10473388
10473389	:	https://www.ncbi.nlm.nih.gov/sra/10473389
10473390	:	https://www.ncbi.nlm.nih.gov/sra/10473390
10473391	:	https://www.ncbi.nlm.nih.gov/sra/10473391
10473392	:	https://www.ncbi.nlm.nih.gov/sra/10473392
10473393	:	https://www.ncbi.nlm.nih.gov/sra/10473393
10473394	:	https://www.ncbi.nlm.nih.gov/sra/10473394
10473395	:	https://www.ncbi.nlm.nih.gov/sra/10473395
10473396	:	https://www.ncbi.nlm.nih.gov/sra/10473396
10473397	:	https://www.ncbi.nlm.nih.gov/sra/10473397
10473398	:	https://www.ncbi.nlm.nih.gov/sra/10473398
10473399	:	https://www.ncbi.nlm.nih.gov/sra/10473399
10473400	:	https://www.ncbi.nlm.nih.gov/sra/10473400
10473401	:	https://www.ncbi.nlm.nih.gov/sra/10473401
10473402	:	https://www.ncbi.nlm.nih.gov/sra/10473402
10473403	:	https://www.ncbi.nlm.nih.gov/sra/10473403
10473404	:	https://www.ncbi.nlm.nih.gov/sra/10473404
10473405	:	https://www.ncbi.nlm.nih.gov/sra/10473405
10473406	:	https://www.ncbi.nlm.nih.gov/sra/10473406
10473407	:	https://www.ncbi.nlm.nih.gov/sra/10473407
10473408	:	https://www.ncbi.nlm.nih.gov/sra/10473408
10473409	:	https://www.ncbi.nlm.nih.gov/sra/10473409
10473410	:	https://www.ncbi.nlm.nih.gov/sra/10473410
10473411	:	https://www.ncbi.nlm.nih.gov/sra/10473411
10473412	:	https://www.ncbi.nlm.nih.gov/sra/10473412
10473413	:	https://www.ncbi.nlm.nih.gov/sra/10473413
10473414	:	https://www.ncbi.nlm.nih.gov/sra/10473414
10473415	:	https://www.ncbi.nlm.nih.gov/sra/10473415
10473416	:	https://www.ncbi.nlm.nih.gov/sra/10473416
10473417	:	https://www.ncbi.nlm.nih.gov/sra/10473417
10473418	:	https://www.ncbi.nlm.nih.gov/sra/10473418
10473419	:	https://www.ncbi.nlm.nih.gov/sra/10473419
10473420	:	https://www.ncbi.nlm.nih.gov/sra/10473420
10473421	:	https://www.ncbi.nlm.nih.gov/sra/10473421
10473422	:	https://www.ncbi.nlm.nih.gov/sra/10473422
10473423	:	https://www.ncbi.nlm.nih.gov/sra/10473423
10473424	:	https://www.ncbi.nlm.nih.gov/sra/10473424
10473425	:	https://www.ncbi.nlm.nih.gov/sra/10473425
10473426	:	https://www.ncbi.nlm.nih.gov/sra/10473426
10473427	:	https://www.ncbi.nlm.nih.gov/sra/10473427
10473428	:	https://www.ncbi.nlm.nih.gov/sra/10473428
10473429	:	https://www.ncbi.nlm.nih.gov/sra/10473429

10473430	:	https://www.ncbi.nlm.nih.gov/sra/10473430
10473431	:	https://www.ncbi.nlm.nih.gov/sra/10473431
10473432	:	https://www.ncbi.nlm.nih.gov/sra/10473432
10473433	:	https://www.ncbi.nlm.nih.gov/sra/10473433
10473434	:	https://www.ncbi.nlm.nih.gov/sra/10473434
10473435	:	https://www.ncbi.nlm.nih.gov/sra/10473435
10473436	:	https://www.ncbi.nlm.nih.gov/sra/10473436
10473437	:	https://www.ncbi.nlm.nih.gov/sra/10473437
10473438	:	https://www.ncbi.nlm.nih.gov/sra/10473438
10473439	:	https://www.ncbi.nlm.nih.gov/sra/10473439
10473440	:	https://www.ncbi.nlm.nih.gov/sra/10473440
10473441	:	https://www.ncbi.nlm.nih.gov/sra/10473441
10473442	:	https://www.ncbi.nlm.nih.gov/sra/10473442
